# A Cross-Sectional Quantitative Metabolomics Study Evidencing the Metabolic Signature in Six Organs during a 14-Week High-Fat High-Sucrose and Standard Diet in Mice

**DOI:** 10.3390/nu16060803

**Published:** 2024-03-12

**Authors:** Eva Drevet Mulard, Sylvie Guibert, Anne Mey, Camille Lefevre, Marie-Agnès Chauvin, Claudie Pinteur, Marie-Ambre Monet, Murielle Godet, Anne-Marie Madec, Béatrice Morio, Jennifer Rieusset, Gilles J. P. Rautureau, Baptiste Panthu

**Affiliations:** 1 ICBMS, CNRS UMR5246,Université Claude Bernard Lyon 1, F-69622 Villeurbanne, France; eva.drevet-mulard@univ-lyon1.fr (E.D.M.); gilles.rautureau@univ-lyon1.fr (G.J.P.R.); 2 Laboratoire CarMeN, UMR INSERM U1060/INRAE U1397, University of Lyon, Université Claude Bernard Lyon 1, F-69310 Pierre-Bénite, Franceca.lefevre@uclouvain.be (C.L.); claudie.pinteur@univ-lyon1.fr (C.P.); marie-ambre.monet@lyon.unicancer.fr (M.-A.M.); anne-marie.coquelet-madec@univ-lyon1.fr (A.-M.M.);

**Keywords:** metabolomics, quantitative metabolomics, NMR, obesogenic diet, metabolite trajectories

## Abstract

Obesity is a risk factor for many diseases, such as type 2 diabetes and cardiovascular diseases. In line with the need for precision medicine, the search for biomarkers reporting the progression of obesity- and diet-associated disorders is urgent. We used NMR to determine the metabolomics profile of key organs (lung, liver, heart, skeletal muscle, kidney, and brain) and serum from male C57Bl/6J mice (5 weeks old) fed for 6, 10, and 14 weeks on a high-fat and high-sucrose diet (HFHSD) vs. a standard diet (STD). We determined metabolite concentrations in the organs at each time point, which allowed us to discriminate age- and diet-related effects as well as the interactions between both, highlighting the need to evaluate the influence of age as a confounding factor on metabolic signatures. Notably, the analysis revealed the influence of time on metabolite concentrations in the STD condition, probably reflecting the juvenile-to-adult transition. Variations impacted the liver and lung metabolites, revealing the strong influence of the HFHS diet on normal metabolism maturation during youth.

## 1. Introduction

Obesity and overweight have reached epidemic proportions worldwide and have nearly tripled since 1975. In 2016, 6.8% of children and adolescents between 5 and 19 years were obese, 39% of adults aged 18 years and over were overweight, and 13.1% were obese [1]. Energy-dense diets, rich in fats and carbohydrates, coupled with reduced physical activity are critical contributors to obesity. Obesity is defined as the excessive expansion of white adipose tissue to counter energy overload by storing it into adipocyte lipid droplets. Dysfunctions or limits to the storage capacity of adipose tissue lead to ectopic fat deposition in non-adipose organs, worsening organ failures [2] and accelerating metabolic complications [3]. This makes obesity one of the most important risk factors for type 2 diabetes and cardiovascular diseases. However, studies have shown that obesity stratified by the body mass index is not indicative of the severity of these risks [4,5], triggering the need for a better understanding of the sequence leading to complications in obesity, notably at the organ level. Metabolic alterations from diet-induced obesity have been well studied, up to the organ level, but most studies were performed using endpoint experiments that compared advanced stages in phenotype development [6,7,8].

Metabolomics studies provide a cutting-edge approach to follow metabolic evolutions over time, resulting from metabolic adjustments and homeostatic constraints. Multiple studies on obese and lean subjects have been recently carried out to compare metabolic profiles of biofluids using metabolomics [9,10,11,12,13,14,15]. The effect of obesogenic diets on metabolism was extensively investigated in mice [16,17] and humans [18], demonstrating significant metabolic adjustments induced by energy-dense diets with serum metabolomic profiles discriminating between obese and lean phenotypes. More recently, the metabolic adaptation of internal organs to the diet has been explored [7,8,17], but the initiation and early progression of the metabolic response associated with a high-energy diet is still poorly documented. Available data mainly report qualitative or relative data and lack the absolute metabolite quantification required for comparisons between studies and for a precise understanding of metabolite alterations. C57Bl/6J mice have long been used for the study of diet-induced obesity and associated metabolic disorders, namely glucose intolerance, followed by insulin resistance and type 2 diabetes [19,20,21,22]. In a previous study, we used proton nuclear magnetic resonance (NMR) spectroscopy to measure metabolite concentrations in internal organs from C57Bl/6J mice fed on a high-fat high-sucrose diet (HFHSD) for 14 weeks, and we revealed organ-specific metabolic signatures associated with obesity [17].

In the present study, we followed the progression of organs’ metabolic signatures over time after 6, 10, or 14 weeks of obesogenic or standard diet in young mice. The metabolite quantifications were performed using NMR in the serum and six key organs in obesity (brain, heart, kidney, liver, lung, and skeletal muscle) to define their endometabolome. NMR spectra allowed the quantification of up to 43 metabolites for each organ. After evidencing organ-specific variations in metabolites over time during the juvenile-to-adult transition, this study reveals the influence of a HFHSD on the trajectory of metabolic changes.

## 2. Materials and Methods

### 2.1. Animal Protocol and Sample Collection

The animal protocol has been described in a previous article [23] and performed in accordance with the institutional guidelines for the care and use of laboratory animals and with the approval of the Regional Committee of Ethics for Animal Experiments. Briefly, 5-week-old C57BL/6J male mice (n = 12/group) (Envigo, Gannat, France) were fed ad libitum for 6, 10, and 14 weeks on a standard (SD, Rhod16A, Genobios, Laval, France) or a high fat-high sucrose diet (HFHSD, 36% *w*/*w* fat, 16.6% *w*/*w* sucrose, Envigo, Gannat, France). One mouse died before 10 weeks of STD, so there were only 11 samples for the 10-week STD groups. At each end-point, retro-orbital blood collection and sacrifice via cervical dislocation were performed on isoflurane-anaesthetized and fed mice. Blood samples were used for serum collection (after a maximum of 1 h on ice, blood samples were centrifuged for 10 min at 5000× *g* and 4 °C). Serum aliquots were stored at −80 °C. Brains, hearts, kidneys, livers, lungs, and muscles were collected, snap-frozen in liquid nitrogen, weighed, and stored at −80 °C. Glucose metabolism was analyzed the week preceding sacrifice using the glucose tolerance test (GTT) and insulin tolerance test (ITT) as described previously [23].

### 2.2. Metabolite Extraction and Sample Preparation for NMR Analysis

Metabolites were extracted as previously described [17]. Briefly, extraction was performed using 100% methanol and a Precellys Homogeniser. The total lysate was put into 5 mL glass tubes, and the Precellys tubes were rinsed with 500 μL of 100% methanol and then dried under a gentle nitrogen flow until complete evaporation. Then, 800 μL of D_2_O phosphate buffer were used to dissolve the dried extracts by vortexing for 30 s. Extracts were then transferred to 1.5 mL Eppendorf tubes and centrifuged at 13,000 rpm for 1 min at 4 °C. Afterwards, 550 μL of supernatant were transferred to 5 mm NMR tubes, and 100 µL of serum were diluted with 100 µL of D_2_O phosphate buffer and directly transferred to 3 mm NMR tubes.

### 2.3. NMR Spectra Analysis

Eight NMR samples delivered poor-quality spectra probably due to the incomplete grinding of the organ samples (one sample from the liver and the brain, four from the kidney, and three from muscle). These outliers were removed from further analysis. 

Lung, liver, heart, skeletal muscle, kidney, and brain extracts and serum NMR spectra were obtained on a Bruker Avance III spectrometer operating at a ^1^H frequency of 800.15 MHz, equipped with a 5 mm TXI probe. The serum samples were measured on a Bruker 600.55 MHz NMR spectrometer equipped with a 5 mm TCI cryoprobe. All spectra were acquired at 303.0° K. The NMR samples were maintained at 4 °C before data acquisition and handled with a Bruker SampleJet automated sample changer. Standard NOESY and CPMG with water presaturation ^1^H 1D NMR spectra were acquired on each sample, with 128 scans for organs or 256 for serum and a spectral width of 20 ppm. For both sequences, the relaxation delay was set to 4 s. A series of 2D NMR experiments (^1^H–^1^H TOCSY, ^1^H–^1^3C HSQC, and J-Resolved) were recorded with standard parameters on a subset of samples to achieve peak assignment of 1D spectra.

### 2.4. Data Processing

The NMR free induction decays (FIDs) were multiplied by an exponential function corresponding to a 0.3 Hz line-broadening factor before Fourier transform. The NMR spectra were referenced using the center of the glucose doublet at 5.230 ppm using Topspin 3.6 (Bruker GmbH, Rheinstetten, Germany). Metabolite identification was obtained using ChenomX NMR Suite 8.0 Software (ChenomX Inc., Edmonton, AB, Canada) and the exploitation of 2D ^1^H–^1^H TOCSY, ^1^H–^1^3C HSQC, and J-Resolved NMR spectra analysis. ChenomX software 8.6 allowed us to determine metabolite concentrations from the ^1^H 1D NOESY experiments. The concentration reference was obtained using a commercial standard lactate solution (1 g/L, Fisher, Waltham, MA, USA), and ERETIC2 utility from TopSpin 3.6.3 (Bruker, RRID:SCR_014227 ) was used to add a synthetic peak to all spectra, enabling cross-referencing [24].

### 2.5. Statistical Analysis

Unsupervised and supervised statistical multivariate analysis were conducted to evaluate sample classification and determine group-specific metabolic signatures. Partial least square discriminant analysis (PLS-DA) [25] was used for sample class discrimination using SIMCA 13 (Umetrics, Umea, Sweden) based on the metabolite concentration variables determined from ChenomX with Pareto scaling. The goodness-of-fit parameters R2 and Q2, which correspond to the explained and predicted variances, respectively, were used to evaluate PLS-DA model performance. Resampling of the models under the null hypothesis was then performed 1000 times to validate the model performance. Analysis of variance (ANOVA) of the cross-validated residuals’ (CV-ANOVA) p-value was also used to assess the significance of the multivariate models [26]. Univariate statistics on metabolite concentrations were obtained by two-way ANOVA (diet and time factors) or one-way (diet factor or time factor, evaluated separately) associated with post hoc *t*-tests [25]. The Benjamini–Yekutieli correction was applied to *p*-values [27]. The data were processed on R 3.6.3 and the graphs by Prism 9.5.0.

## 3. Results

### 3.1. Experimental Design

#### 3.1.1. Phenotype of the HFHSD-Induced Obese Mice

The experimental protocol in mice was designed to observe changes induced by obesity from 6 weeks of diet, and before the emergence of systemic inflammation, to focus on the effects directly related to metabolic changes [23]. In addition to whole body and adipose tissues weight gains, glucose intolerance was observed after 5 and 9 weeks of HFHSD and resistance to insulin after 13 weeks, illustrating the progressive alteration of the glucose metabolism by the HFHSD [23]. Except for the liver, of which weight increased significantly with obesity from 1.4 g in control mice to 2 g and 2.8 g after, respectively, 10 and 14 weeks of HFHSD (Supplementary Figure S1), the weight of the other organs analyzed in this study was not changed. 

#### 3.1.2. NMR Determination of Organ and Serum Metabolic Profiles

**Figure 1:**
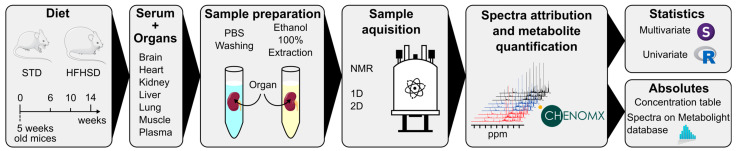
Schematics presenting the principal steps conducted to determine metabolic signature induced by the obesogenic diet.

To determine the metabolic profiles in organs, we used NMR spectroscopy to acquire spectra from organ extracts and serum of STD and HFHSD mice, after 6, 10, and 14 weeks of diet (n = 11–12 per group). We determined the absolute concentration of series of metabolites, allowing a temporal comparison of HFHSD vs. STD metabolic profiles in several organs. The organ extracts and the serum delivered well-resolved NMR spectra that displayed sharp peaks typical of small molecules, overlaid with broad signals originating mostly from lipids in organ extracts or lipids plus proteins in the serum. The identification and quantification of the metabolites were performed using NMR spectra as previously described in [17]. In total, the concentration of up to 43 metabolites was determined in six organs from up to 12 mice analyzed individually at the three kinetic end-points. The workflow is presented in Figure 1.

### 3.2. HFHSD-Induced Metabolic Alterations

**Figure 2:**
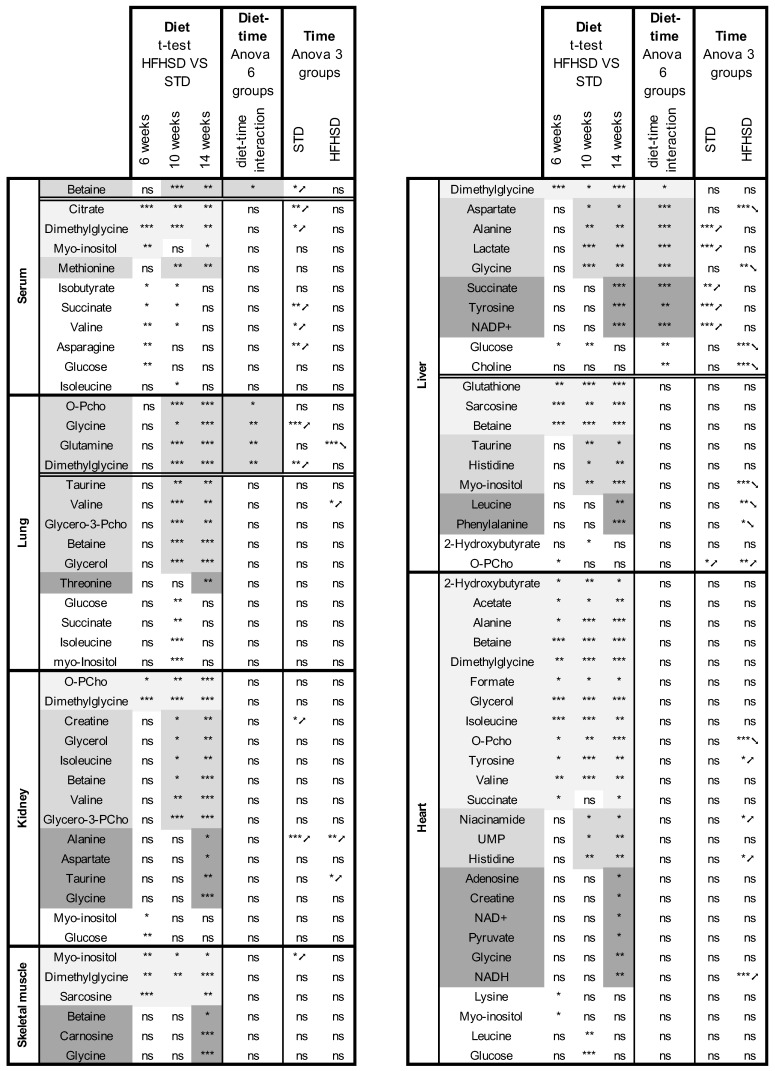
It summarizing the statistical analysis of the impact of diet over time on mice organs and serum. Only metabolites that present a significant impact of diet with ANOVA and *t*-tests are reported. Columns 1 to 3 report comparisons by pairs using post hoc *t*-tests (6 weeks STD vs. HFHSD, 10 weeks STD vs. HFHSD, and 14 weeks STD vs. HFHSD). Column 4 reports the results for the interaction between diet duration (time) and composition (diet) from a two-way ANOVA analysis. The color code (greyscale) classifies the appearance of statistical difference as early (appearance at 6 weeks, light grey), median (appearance at 10 weeks, medium grey), and late (appearance at 14 weeks, dark grey). Columns 5 and 6 report the results from one-way (time) ANOVA testing over the 3 groups of mice (6, 10, and 14 weeks) for the STD (column 5) and HFHSD group (column 6). The evolution over time is described by the arrows as follows: upward for increasing, downward for decreasing. Results are indicated as non-significant (ns), *p* < 0.05 (*), *p* < 0.005 (**), and *p* < 0.0005 (***). Data were corrected for multiple testing (Benjamini–Yekutieli).

Using PLS-DA supervised multivariate analysis, we were able to distinguish the metabolite signature between HFHSD and STD mice in all organs and the serum after 14 weeks of diet, except the brain (Supplementary Figure S2). Metabolite signatures from multivariate analyses evidenced organ-specific adaptation to HFHSD early in obesity; similar observations were made at the unique end-point of 14 weeks of the diet in our previous study [17]. Performing ANOVA and *t*-test analyses to compare individual metabolite concentrations from STD and HFHSD fed mice in each organ, and at each time, we confirmed diet-specific variations affecting metabolite concentrations. These results also revealed a distinct sensitivity of organs to HFHSD exposure with, at both extremes, the heart, having a majority of metabolite concentrations already altered after 6 weeks of diet, and the lung, where differences appeared after 10 weeks of diet. The other organs presented intermediate situations, as a function of the precocity of metabolites concerned. The results are presented in the first three columns of Figure 2. The heart and the liver were the most impacted organs, with 25 and 19 metabolites altered out of 40 and 43 quantified metabolites, respectively. Betaine, DMG, and glycine were the most impacted in the liver. Conversely, the brain and the serum were less affected with 0 and 11 metabolites altered out of 40 and 39 metabolites. Despite these differences, some metabolite variations were shared between five organs (heart, kidney, liver, lung, and muscle) and with serum. This was the case for betaine, DMG, and myo-inositol. Of interest, glucose was altered in all organs, except in the muscle and the brain. Glycine and o-phosphocholine were affected in all organs except in the serum. Some metabolite modifications were specific to a single organ. For instance, glutathione and lactate were significantly impacted in the liver only. Adenosine, formate, NADH, niacinamide, and UMP were impacted in the heart but not in the other organs. Likewise, glutamine and threonine were specific to the lungs; carnosine, glucose-1-phosphate, glucose-6-phosphate, and mannose were specific to muscle; and asparagine, citrate, isobutyrate, and methionine were specific to the serum. No metabolite variation was specific to the kidney.

Examining in more detail the source of variations between STD and HFHSD, we observed that metabolite concentrations in the STD condition vary with time, as shown by the PLS-DA supervised multivariate analysis on each organ (Supplementary Figure S3). To determine whether diet and time interact with each other to influence individual metabolite concentrations, we performed a two-way ANOVA. The results in column 4 of Figure 2 showed significant interactions between both variables mostly in the lung and in the liver with several metabolites involved. In the serum, only betaine was subjected to the double influence of diet composition and duration, and no interactions were detected in the other organs where variations were mostly attributed to the composition of the HFHSD.

To confirm the influence of diet duration on metabolite concentration, we performed one-way ANOVA, comparing the concentration of each metabolite after 6, 10, and 14 weeks of either STD or HFHSD. The results are presented in columns 5 and 6 of Figure 2. The red overlining designed metabolite concentrations increasing with time while the green color indicated decreasing metabolite concentrations. The six group boxplots (STD and HFHSD, at 6, 10, and, 14 weeks) for each metabolite and in each organ are presented in Supplementary Figure S4. Most of the variations in time affected either the STD condition or the HFHSD condition and reflect the influence of the age in this protocol starting in prepubescent male mice (5 weeks old) with analysis performed at the ages of 11, 15, and 19 weeks [28].

### 3.3. Time-Related Metabolite Evolution over 14 Weeks of Diet

**Figure 3:**
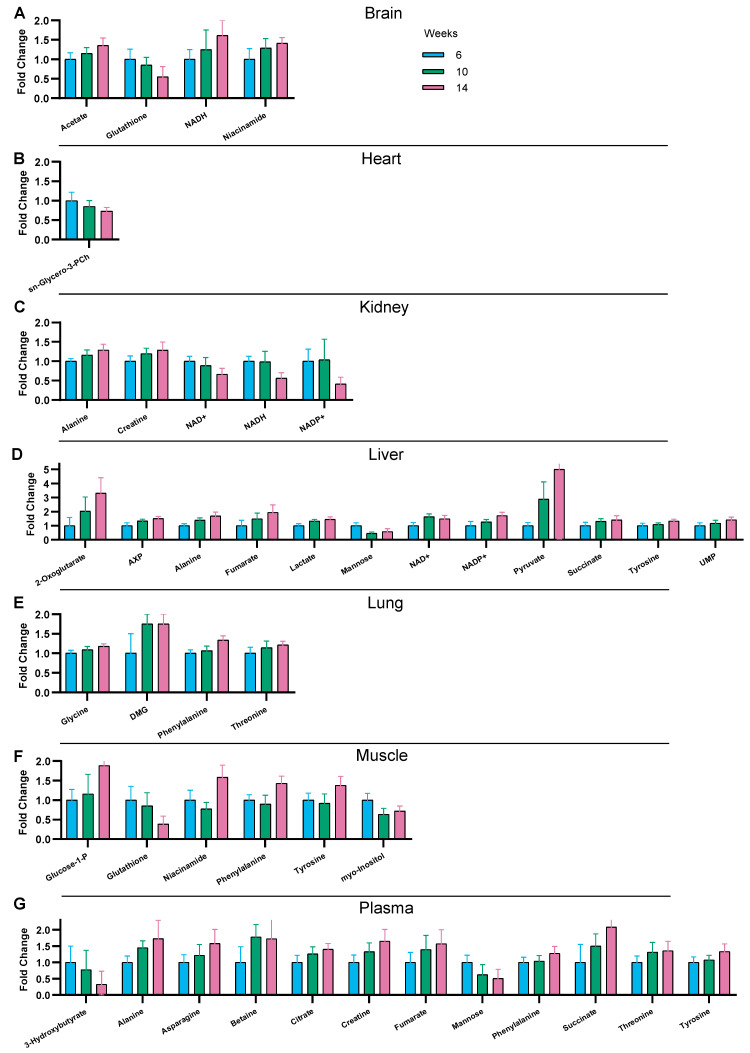
Comparison of the relative concentration of metabolites for the STD groups at weeks 6, 10, and 14 (in blue, green, and pink, respectively) in organs and serum. Only metabolites that present a significant interaction with time as tested by one-way ANOVA are presented. For readability, data are presented relative to the metabolite level at 6 weeks (the tables of concentrations are presented in supplementary materials). The histogram indicated the mean ± SD (n = 10 to 12 depending on the organs). (**A**) Brain; (**B**) Heart; (**C**) Kidney; (**D**) Liver; (**E**) Lung; (**F**) Muscle; (**G**) Plasma.

Aging over 14 weeks alone, independently of other factors, impacts the physiological status of animals and the metabolite levels [29]. Multivariate statistical analysis by partial least squares-discriminant analysis (PLS-DA) of the metabolic fingerprint of the organs were first used to evaluate potential group discrimination between STD and HFD conditions after 6, 10, and 14 weeks of diet. In the six organs and the serum, time appeared as a discriminating variable in control mice (Supplementary Figure S3) reflecting the normal evolution of the organs during the timescale of our study. This was confirmed by one-way ANOVA on individual metabolites (Figure 2, columns 5 and 6), indicating a constant progression of the concentration with time, despite non-significant differences when comparing time points by pairs (6 vs. 10 or 10 vs. 14 weeks). Variations in metabolite concentrations in the STD group over time are represented in Figure 3, presenting for each organ the metabolites showing the significant influence of time in ANOVA. The serum and liver displayed a higher number of metabolites altered with time, while the heart, lung, and brain were less affected by the juvenile-to-adult transition. Interestingly, no common metabolite varied over time between the six organs and the serum. The liver and serum shared alterations in alanine, fumarate, mannose, succinate, and tyrosine levels. Importantly, some metabolites were impacted in only one organ, reflecting unique organ-dependent changes in metabolism over time. For instance, the liver presented the following six unique metabolites impacted, which varied between 6 and 14 weeks: 2-oxoglutarate, AXP, lactate, o-phosphocholine, pyruvate, and UMP. The concentration of glycine was significantly altered only in the lung, sn-glycero-3-phosphocholine in the heart, glucose-1-phosphate in muscle, and acetate in the brain. Finally, the serum had five specific metabolite markers related to the juvenile-to-adult transition: 3-hydroxybutyrate, asparagine, betaine, citrate, and valine. Using one-way ANOVA, a significant influence of time on metabolite concentrations was also observed with HFHSD. Most of these changes were not observed with the STD, indicating the influence of factors distinct from the normal aging of organs. This may reflect alterations related to the immaturity of the mice at the beginning of the protocol with obesity starting in 5 weeks-old mice.

### 3.4. Interaction between Diet and Time in the Juvenile-to-Adult Transition

We assessed the impact of time and type of diet for each metabolite and organ using two-way ANOVA. The boxplots for the individual metabolite concentrations are presented in Supplementary Figure S4, for the six groups (HFHSD and STD, at 6, 10, and 14 weeks). Two-way ANOVA evidenced a significant interaction between diet duration and diet composition. This was mainly observed in the liver, followed by the lung and, to a lesser extent, the serum (Figure 2, column 4). In the liver, AXP, acetone, alanine, aspartate, choline, glucose, glutamine, glycine, lactate, lysine, NADP+, DMG, succinate, tyrosine, UMP, and sn-glycero-3-phosphocholine presented a diet–time interaction. In the lungs, glutamine, glycine, DMG, and o-phosphocholine showed diet–time interactions. In the serum, betaine was the only metabolite presenting an interaction between diet and time. On the contrary, there was no metabolite showing interactions between time and diet in the heart, kidney, muscle, and brain. Interactions were associated with a significant influence of time either in the STD or in the HFHSD condition but not both for a given metabolite, inducing variations that increase or decrease with time. The biological significance of this interaction remains to be determined, but these results point out the importance of duration in addition to the type of diet for the analysis of individual metabolite variations with obesity. This reflects metabolic regulations normally evolving with age and that are altered by obesity.

## 4. Discussion

**Figure 4:**
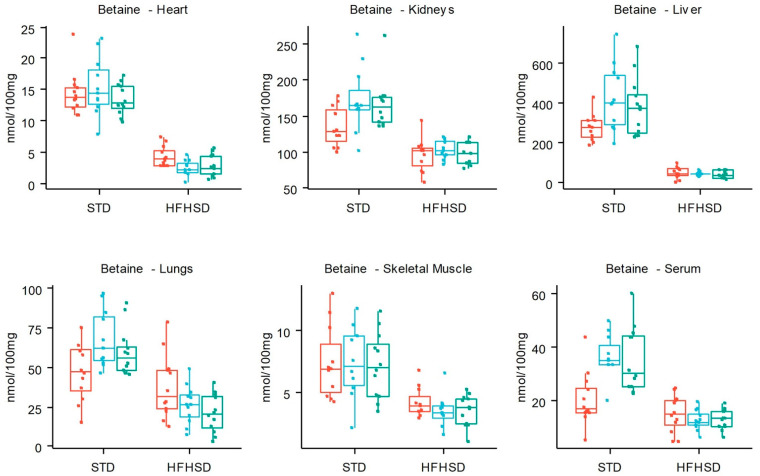
Betaine concentration in nmol/100 mg of organ, depending on the diet (STD and HFHSD) and over time (6, 10, and 14 weeks, respectively, in red, blue, and green), for each organ except the brain. The 3 horizontal bars represent the median, the first, and the third quartile of the boxplots. The associated statistics are available in Figure 2.

The development of obesity is a progressive process affecting organs and systemic metabolisms. Measuring metabolite concentrations may represent a good way to assess health status and disease progression. From a previous study performed in mice fed a STD or a HFHSD for 14 weeks, we demonstrated obesity-induced changes in metabolite concentrations measured by NMR in serum and organs [17]. However, the details of metabolite changes over time at the local or systemic level were not known, impairing the interpretation of variations that affect metabolite concentrations. To gather further insights in this matter, in the present study, we followed the metabolite concentrations in the serum and in five organs after 6, 10, and 14 weeks of HFHSD compared to STD. We included the measurement of new metabolites such as AXP, NAD+, NADH, and NADP+. Only the heart NADH and NAD and the liver NADP+ concentrations were affected by 14 weeks of HFHSD. We confirm, in this independent study, that the brain was the least affected by obesity with no significant change detected, but, in all the other organs and the serum, metabolite concentrations presented changes ranging up to 80% concentration variation. In accordance with our first study’s observations, lung metabolites were strongly impacted by HFHSD with significant differences confirmed for seven out of the nine already reported alterations (betaine, glutamine, myo-inositol, o-phosphocholine, sn-glycero-3-phosphocholine, taurine, and valine) after 14 weeks of diet. The exceptions were phenylalanine with no significant difference reported, and serine, which could not be quantified in this study. Threonine, which was not quantified in the first study, was significantly impacted here. We also confirm higher concentrations of glucose with HFHSD in the serum and all the organs except the muscle. This glucose increase reflects homeostatic changes associated with the fed state of mice at the time of organ collection and were in accordance with the intolerance to glucose observed at all time points examined using the GTT in fasted mice [23]. The low glucose concentration in the muscles is probably due to its known rapid processing in this organ during the metabolite extraction process [30]. Another observation of our previous study was the significant increase in citrate and succinate concentrations with HFHSD in the kidney and in the serum but not in any other organ, reflecting alterations in TCA-derived metabolites by diet with potential consequences in providing energy and acetyl groups. Only the increase in citrate concentration in the serum was reproduced here, with succinate displaying a non-significant tendency. Importantly, our previous study also evidenced a major alteration in the one carbon (1C) metabolism by HFHSD detected in all organs and the serum. This was confirmed here with an important HFHSD-induced decrease in the methylated forms of glycine, namely methyl-glycine (betaine), dimethyl-glycine (DMG), and trimethyl-glycine (sarcosine) in serum and all organs except the brain, as illustrated in Figure 4 for the betaine. These methyl-donor metabolites belong to the 1C metabolism pathway that appeared sensitive and adaptable to the diet with possible consequences on epigenetic regulations and post-translational modifications not investigated here. Therefore, this study confirmed that (i) the measurement of metabolite concentrations reflects the adaptation of the organs to obesity, (ii) changes affecting metabolites of the 1C metabolism are common features of the serum and all the organs tested, except the brain, (iii) metabolite changes affecting the lung are highly reproducible between our two studies, and (iv) the measurement of citrate in the serum is a reproducible marker of mitochondrial activity as we recently shown in ex vivo studies [31].

The second point examined here is the delay of the onset of alterations affecting metabolite concentrations after the beginning of the HFHSD. We compared 6 and 10 weeks of diet-induced glucose intolerance, with 14 weeks of diet where insulin resistance has arisen, reflecting the progression toward complications that are associated with the body weight gain [23]. A prominent observation is that the early onset of HFHSD-induced metabolite changes varies between organs. Interestingly, the metabolic pathways targeted by HFHSD in both studies were also the earliest with alterations already observed at 6 weeks of diet. This was not only the case for the concentration of the TCA intermediate citrate in serum but also for most of the metabolites of the 1C metabolism with differences between organs. This may reflect an adaptation of the TCA and 1C metabolisms to primary nutrition and obesity rather than to secondary factors subsequent to organ alterations. While the liver appeared as the most responsive organ to obesity by the number of metabolites impacted, only 5 out of 17 were already altered after 6 weeks of diet. Differently, in the heart, 14 out of 23 metabolites were early responders to obesity. In the lung, where the identity of the metabolites altered after 14 weeks of diet was well conserved between our both studies, no early responder was found, and changes mostly occurred after 10 weeks of diet (9 metabolites out of 10). Metabolite modifications that arose after 14 weeks of HFHSD could be a consequence of resistance to insulin and may reflect installed metabolic adaptation to obesity. Further investigations using longer diet exposures would be required to establish the link between metabolite disturbances and their biological origins.

**Figure 5:**
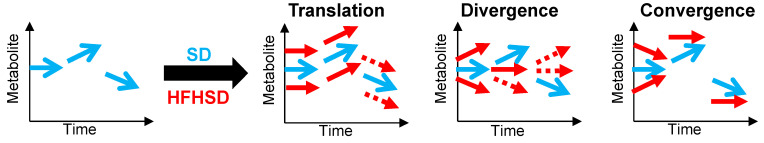
A model of metabolite trajectories according to a standard diet (SD, blue arrows)), where metabolite concentration can be stable, increase, or decrease over time, and trajectories changes after a high-fat and high-sucrose diet (HFHSD, red arrows) over time. Dashed arrows indicate metabolite trajectories that were not observed in this study. The translation (or parallel) model suggests a homeostatic readjustment that occurs early during diet modification (before 6 weeks of diet in our design of experience). Sarcosine, betaine, and dimethyl-glycine in the liver illustrate the translation model (see Supplementary Figure S4). The divergence model indicates relevant metabolites that could become key biomarkers throughout life. Longer observation time would make them significant, and an absolute concentration threshold of the metabolite could serve as a reference. Leucine and phenylalanine in the liver well illustrate the divergent model (see Supplementary Figure S4). In contrast, the convergent model indicates metabolites that would lose their relevance according to the metabolic flexibilities or natural metabolic trajectories. The asparagine and citrate in serum well illustrate the convergent model (see Supplementary Figure S4).

Another result from this study is the evidence of the impact of diet on the time evolution of some metabolite concentrations. Whether the concentration of one metabolite over time in the HFHSD condition paralleled or not, the concentration in the STD condition may lead to distinct interpretations, particularly if time also affected the concentration in the STD condition. Indeed, in all the organs except the heart and the serum, the concentration of some metabolites was significantly different between 6 weeks and longer time of diet, suggesting the influence of age independent of diet (Figure 2, column 3). This may reflect the juvenile-to-adult transition between mice aged 11 weeks old and mice aged 15 or 19 weeks old at the end of the protocol. Several metabolites reached levels after 6 weeks of HSHSD normally obtained in 15- or 19-week-old lean mice. This was observed with asparagine, citrate, succinate, and valine in serum; alanine in kidney; and myo-inositol in muscle (Supplementary Figure S4). In this case, there was a convergence between the STD and the HFHSD metabolite concentrations over time, and differences were restricted to the shorter time exposures. For some metabolites, diet duration similarly affected both diet conditions, giving translation (i.e., lower parallel or higher parallel) evolutions with time and differences between both diets were maintained over time. A last encountered situation was the divergence where time affected only the metabolite concentration in the HFHSD condition, indicating obesity-induced alterations that were not reproduced by aging. The evolutions of the translation, convergence, and divergence of individual metabolite concentrations over time in relation to the influence of the diet are illustrated in Figure 5. They evidenced age subgroups in all organs identified by distinct concentrations of metabolites. 

Several aging studies reported metabolite changes, mainly on longer timescales than in this work, usually using mice aged between 10 and 9 weeks. These studies [32,33,34,35,36,37], converge towards several aging biomarkers. Among them, lactate and citrate were proposed as aging-specific biomarkers in tissues, and glucose in serum [36]. In our study, age only influenced the citrate level. Tyrosine was also proposed as a main biomarker of aging in the brain, heart, kidney, liver, lung, and spleen [35]. In our experimental conditions, tyrosine was impacted in the liver, muscle, and serum but not in the lung, brain, heart, and kidney. Our study focused on a much shorter window of time and younger mice than what was studied elsewhere. Indeed, between 11 and 19 weeks of age examined here, adolescent mice reach adulthood and become sexually active [28]. The data provided here partially confirm the metabolites already reported to vary with aging. We also identified additional metabolites related to earlier biological maturation or development with organ specificity. For instance, we identified six organ-specific metabolites in the liver (2-oxoglutarate, AXP, lactate, O-phosphocholine, pyruvate, and UMP), five in the serum (3-hydroxybutyrate, asparagine, betaine, citrate, and valine) and only one in the lungs (glycine), heart (sn-glycero-3-phosphocholine), muscle (glucose-1-phosphate), and brain (acetate). They reflect organ-dependent changes in metabolism over time during the juvenile-to-adult transition. If validated in further studies, these metabolite targets could help study the impact of organ-targeting or anti-aging drugs. 

## 5. Conclusions

Using NMR, this study provides absolute concentration tables describing the evolution of individual metabolites in five organs and serum in response to HFHSD in a non-targeted way. Such data allow a more precise classification of candidate biomarkers, enabling us to include the time- or aging-related variation dimension in the description of the reference biomarker levels, for example. This study outlines the relevance of NMR and the kinetic approach for the characterization of the model and identification of suitable biomarkers that can next be measured using other analytical methods for targeted metabolite quantification.

The absolute concentration tables of metabolites in mice organs and serum, determined from NMR analyses, represent a reference dataset for further studies of diet-induced obesity investigations in young mice.

## Data Availability

The datasets used and/or analyzed during the current study are available from the corresponding author upon reasonable request.

## Supplementary Materials

The following supporting information can be downloaded at: https://www.mdpi.com/article/10.3390/nu16060803/s1. Table S1: The average and standard deviation of the concentrations in nmol/100g are presented in the following sheets for each organ and in µmol/L for the serum. Figure S1. Organ weight gains. Figure S2. NMR-based metabolomics analyses of SD-mice allow to evidence the impact of juvenile to adult transition on individual organs and serum. Figure S3. The supervised PLS-DA models based on 43 metabolite concentrations allow to evidence the impact of the diet at 14 weeks (HFHSD vs SD, in grey and black respectively) on individual organs and serum.Figure S4. Metabolite concentrations in nmol/100 mg of organ, depending on the diet (STD and HFHSD) and over time
