# Supplementary material for: A Cross-Sectional Quantitative Metabolomics Study Evidencing the Metabolic Signature in Six Organs during a 14-Week High-Fat High-Sucrose and Standard Diet in Mice
Source: Nutrients. 2024 Mar 12;16(6):803. doi: 10.3390/nu16060803 (PMC10975117; doi:10.3390/nu16060803)
Supplement: Supplementary file 1 [file nutrients-16-00803-s001.zip › nutrients-2853694-supplyment figures.pdf]

nutrients

MDPI

*Article*

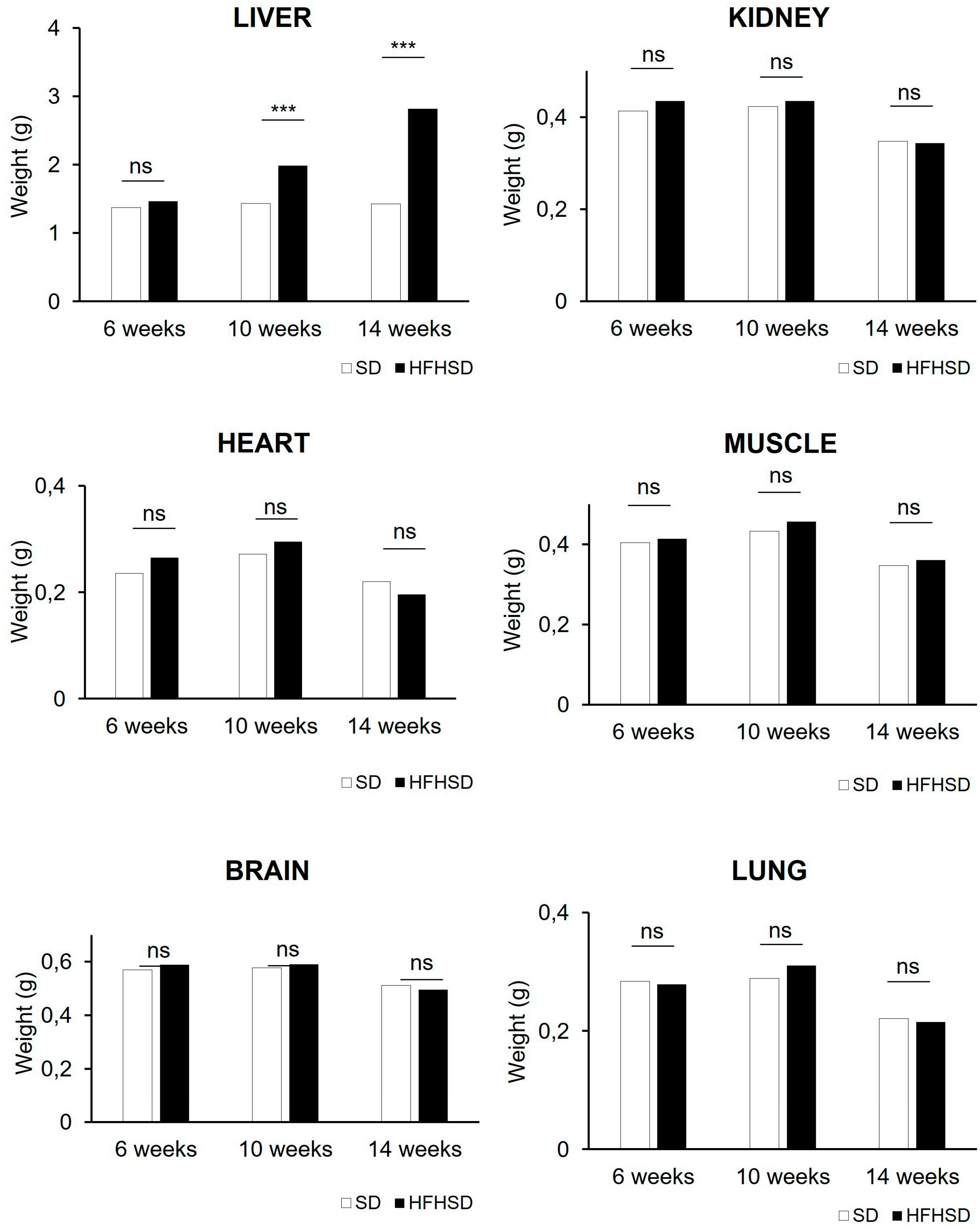


**Supplementary Figure 1.** Organ weight gains, mean +/- SD from n = 12 mice per group. Ns: non significative, ***: p<0.001.

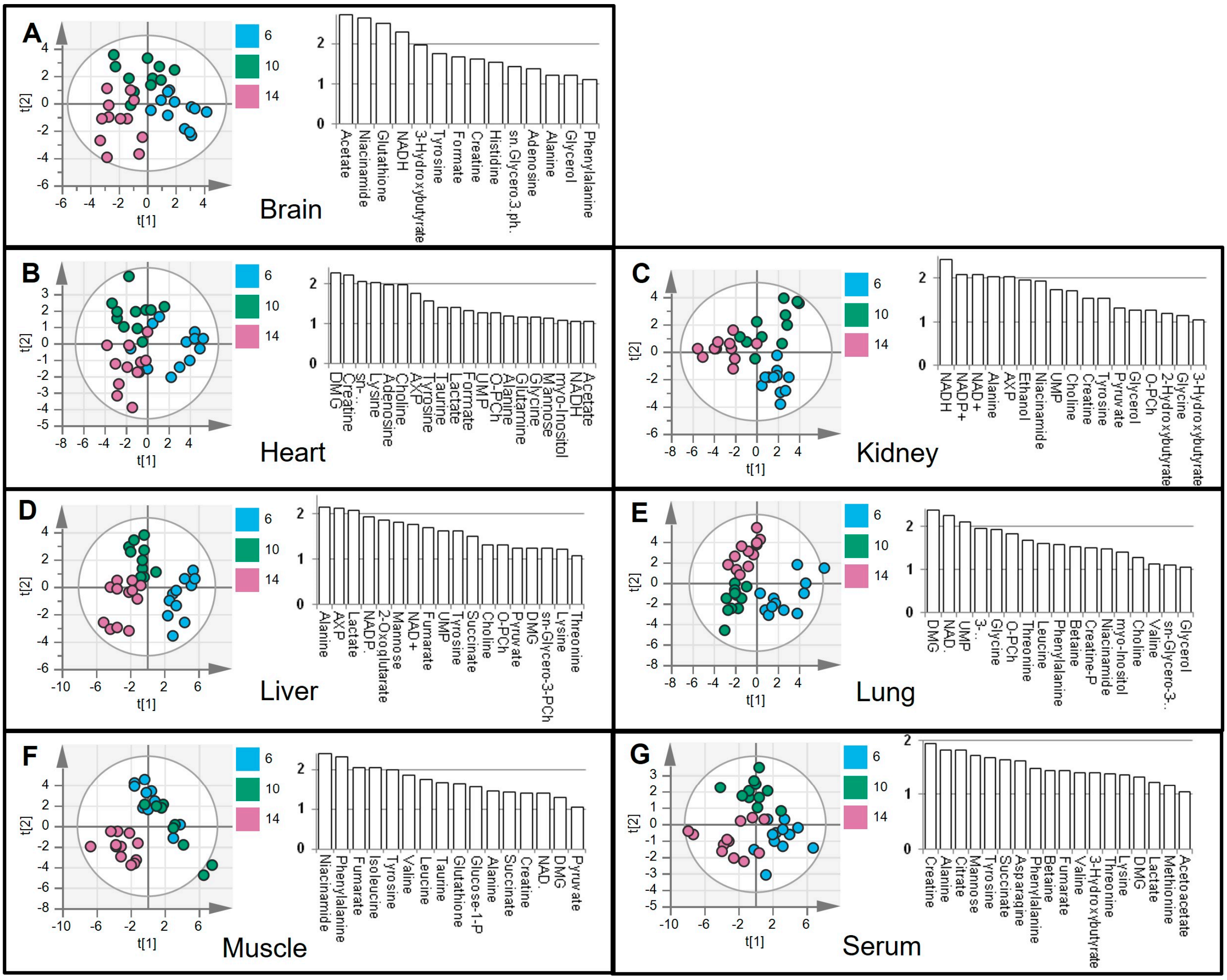


**Supplementary Figure 2.** NMR-based metabolomics analyses of SD-mice allow to evidence the impact of juvenile to adult transition on individual organs and serum. The supervised PLS-DA models based on 43 metabolite concentrations derived from 1H NMR profiles show the discrimination of the different time groups (6, 10 and 14 weeks on 5 weeks old mice). The score plots for each organ are presented. They present sample projections onto the predictive axis and the first orthogonal component of the model. The variable importance values in the projection (VIP) for each model are also presented. (A) Brain: score plot of the PLS-DA model (n=35, 2 components, R2(X) = 0.243, R2(Y) = 0.628, and Q2 = 0.406; CV-ANOVA p = 2.3 × 103). (B) Heart: score plot of the PLS-DA model (n=35, 2 components, R2(X) = 0.278, R2(Y) = 0.593, and Q2 = 0.305; CV-ANOVA p = 1.3 × 103). (C) Kidney: score plot of the PLS-DA model (n=34, 2 components, R2(X) = 0.300, R2(Y) = 0.717, and Q2 = 0.510; CV-ANOVA p = 9.4 × 106). (D) Liver: score plot of the PLS-DA model (n= 34, 3 components, R2(X) = 0.432, R2(Y) = 0.814 and Q2 = 0.605; CV-ANOVA p = 4.2 × 106). (E) Lung: score plot of the (2 + 1) PLS-DA model (n=35, 3 components, R2(X) = 0.458, R2(Y) = 0.838, and Q2 = 0.662; CV-ANOVA p = 1.7 × 102). (F) Muscle: score plot of the (2 + 2) PLS-DA model (n=33, 4 components, R2(X) = 0.664, R2(Y) = 0.880, and Q2 = 0.732; CV-ANOVA p = 5.0 × 107). (G) Serum: score plot of the (2 + 1) PLS-DA model (n=35, 3 components, R2(X) = 0.574, R2(Y) = 0.751, and Q2 = 0.588; CV-ANOVA p = 1.1 × 106).

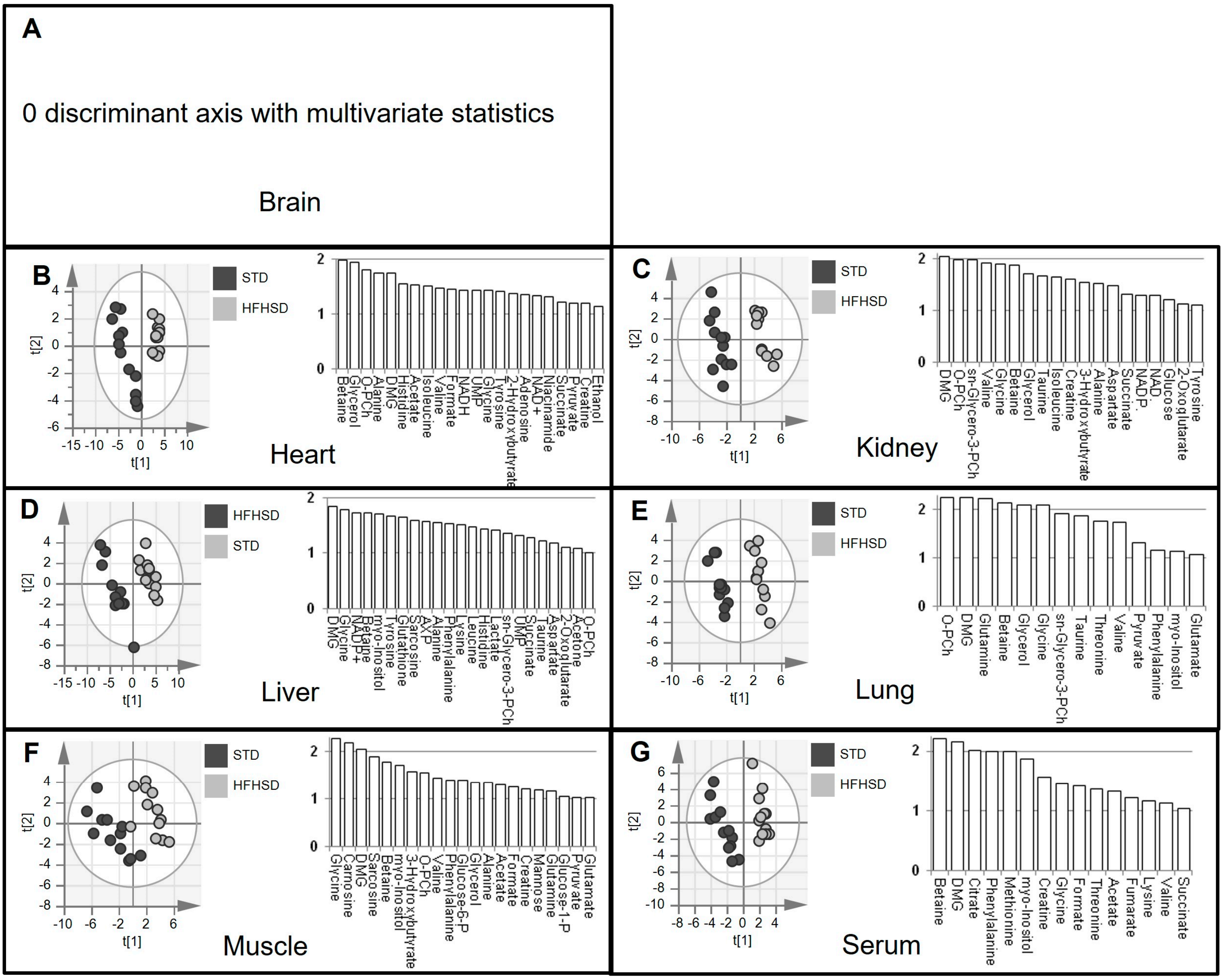


**Supplementary Figure 3.** The supervised PLS-DA models based on 43 metabolite concentrations allow to evidence the impact of the diet at 14 weeks (HFHSD vs SD, in grey and black respectively) on individual organs and serum. The PLS-DA score plots are presented for each organ. They present sample projections onto the predictive axis and the first component of the model. The variable importance values in the projection (VIP) for each model are also presented. (A) Brain: No significant discrimination model could be obtained. (B) Heart: score plot of the PLS-DA model (n=24, 3 components, R2(X) = 0.619, R2(Y) = 0.977, and Q2 = 0.923; CV-ANOVA p = 9.1 × 108). (C) Kidney: score plot of the PLS-DA model (n=23, 2 components, R2(X) = 0.445, R2(Y) = 0.959, and Q2 = 0.904; CV-ANOVA p = 9.8 × 109). (D) Liver: score plot of the PLS-DA model (n= 24, 2 components, R2(X) = 0.965, R2(Y) = 0.924 and Q2 = 0.605; CV-ANOVA p = 2.9 × 1010). (E) Lung: score plot of the PLS-DA model (n=24, 3 components, R2(X) = 0.471, R2(Y) = 0.989, and Q2 = 0.941; CV-ANOVA p = 8.9 × 109). (F) Muscle: score plot of the model (n=24, 2 components, R2(X) = 0.500, R2(Y) = 0.854, and Q2 = 0.781; CV-ANOVA p = 1.1 × 105). (G) Serum: score plot of the PLS-DA model (n=24, 2 components, R2(X) = 0.504, R2(Y) = 0.953 and Q2 = 0.907; CV-ANOVA p = 5.8 × 109).

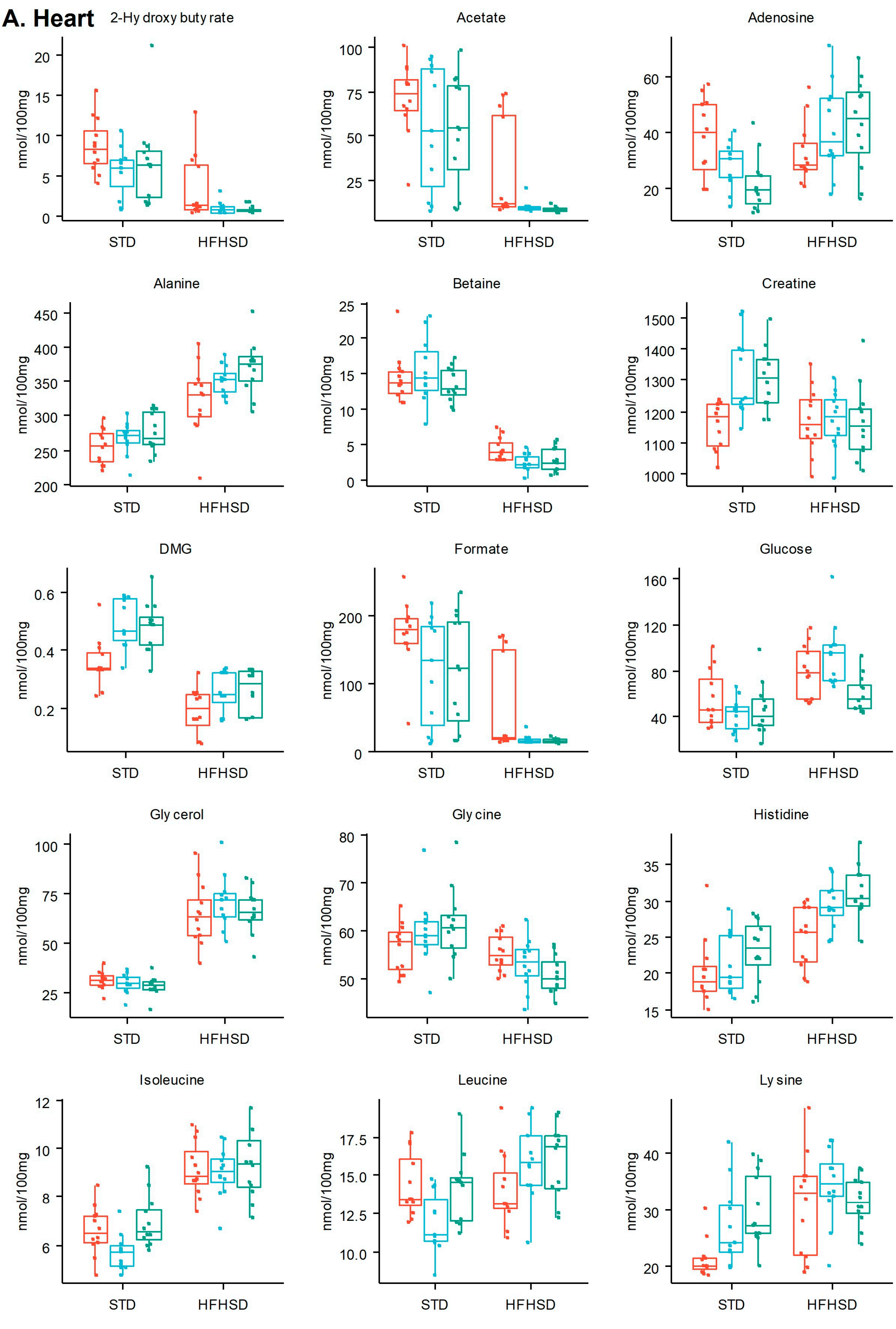
A. Heart
2-Hy droxy buty rate
Acetate
Adenosine
Alanine
Betaine
Creatine
DMG
Formate
Glucose
Gly cerol
Gly cine
Histidine
Isoleucine
Leucine
Ly sine
nmol/100mg
STD
HFHSD

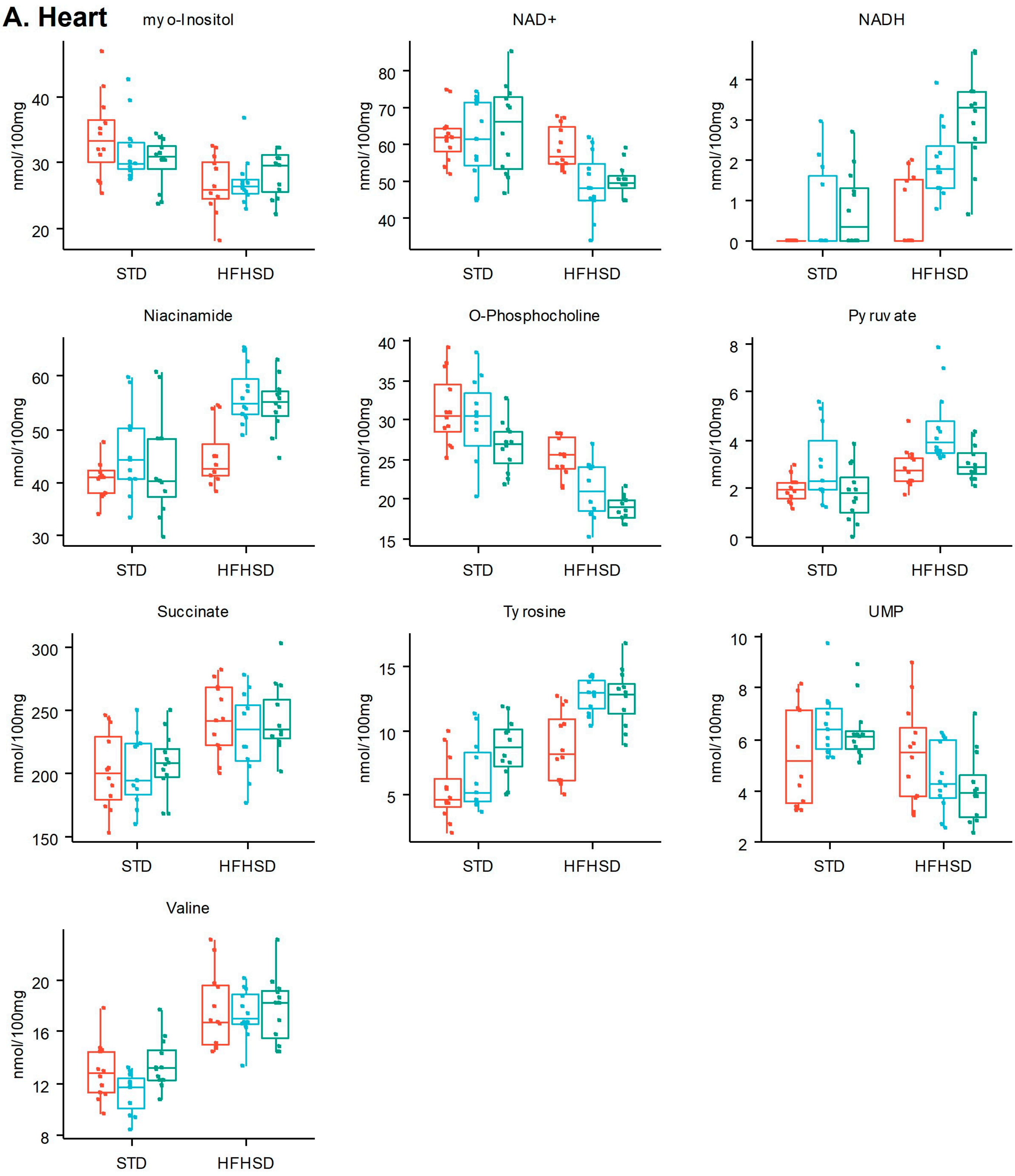

A. Heart
my o-Inositol
NAD+
NADH
Niacinamide
O-Phosphocholine
Py ruv ate
Succinate
Ty rosine
UMP
Valine
nmol/100mg
STD
HFHSD

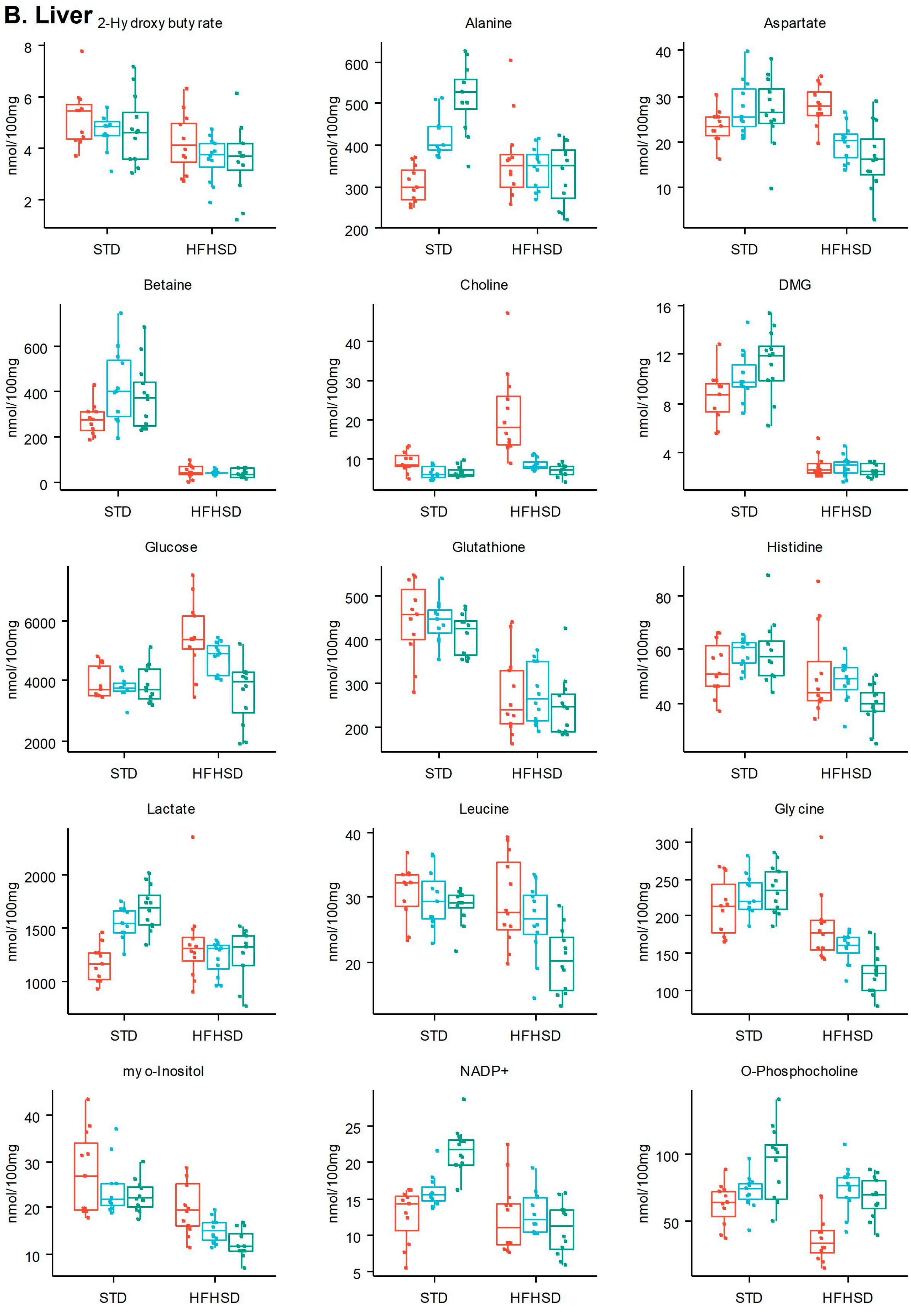
B. Liver
2-Hy droxy buty rate
Alanine
Aspartate
Betaine
Choline
DMG
Glucose
Glutathione
Histidine
Lactate
Leucine
Gly cine
my o-Inositol
NADP+
O-Phosphocholine
nmol/100mg
STD
HFHSD

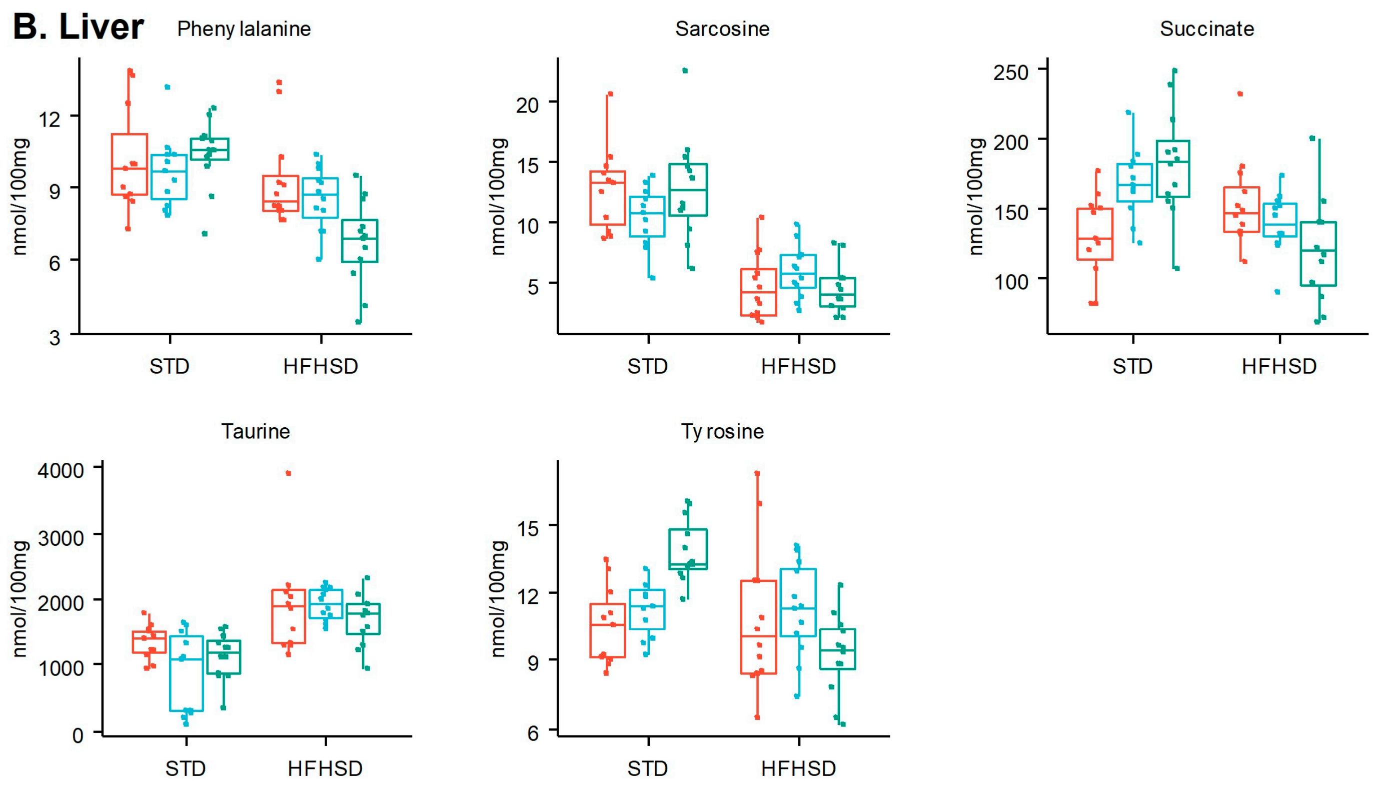
B. Liver
Pheny lalanine
Sarcosine
Succinate
Taurine
Ty rosine
nmol/100mg
STD
HFHSD

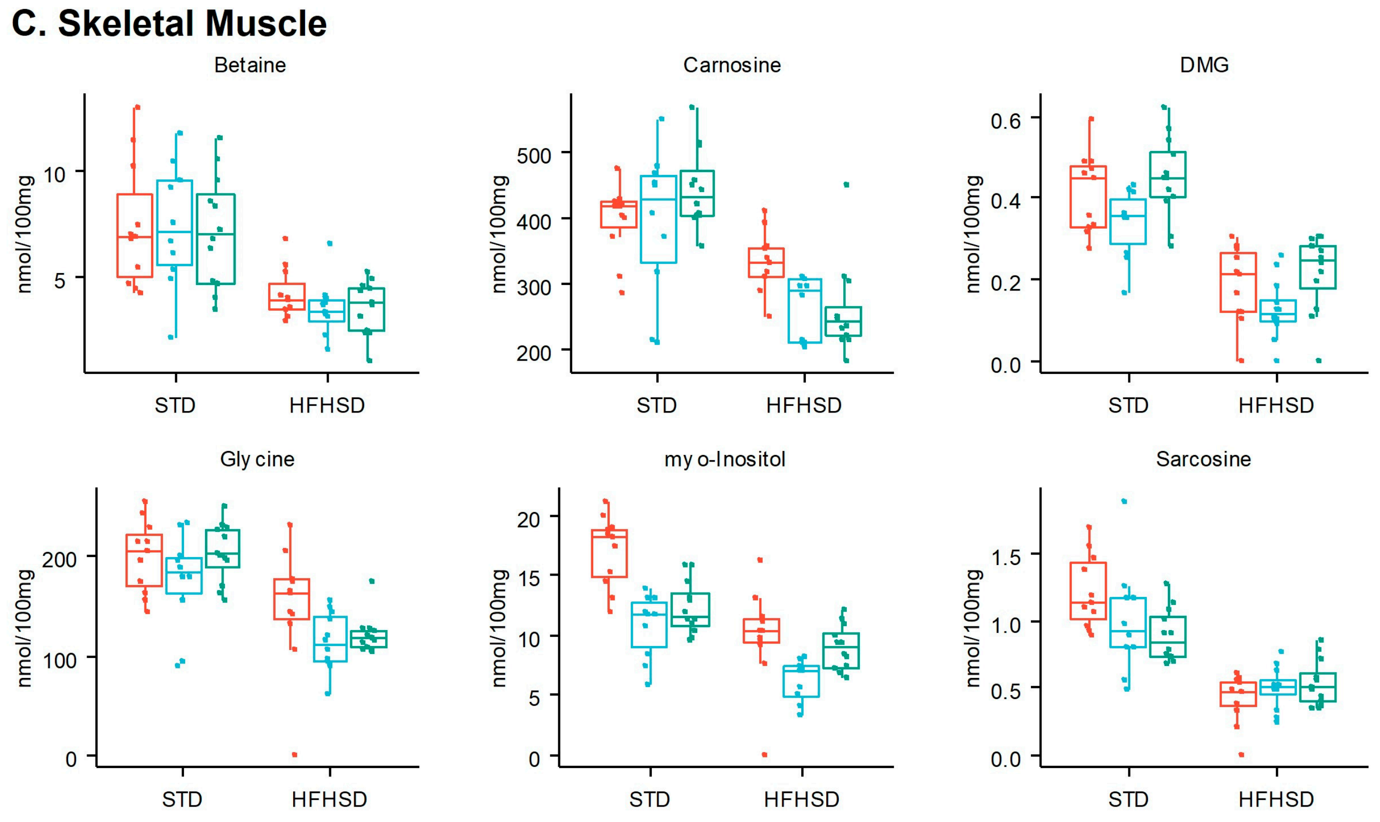
C. Skeletal Muscle
Betaine
Carnosine
DMG
Gly cine
my o-Inositol
Sarcosine
nmol/100mg
STD
HFHSD

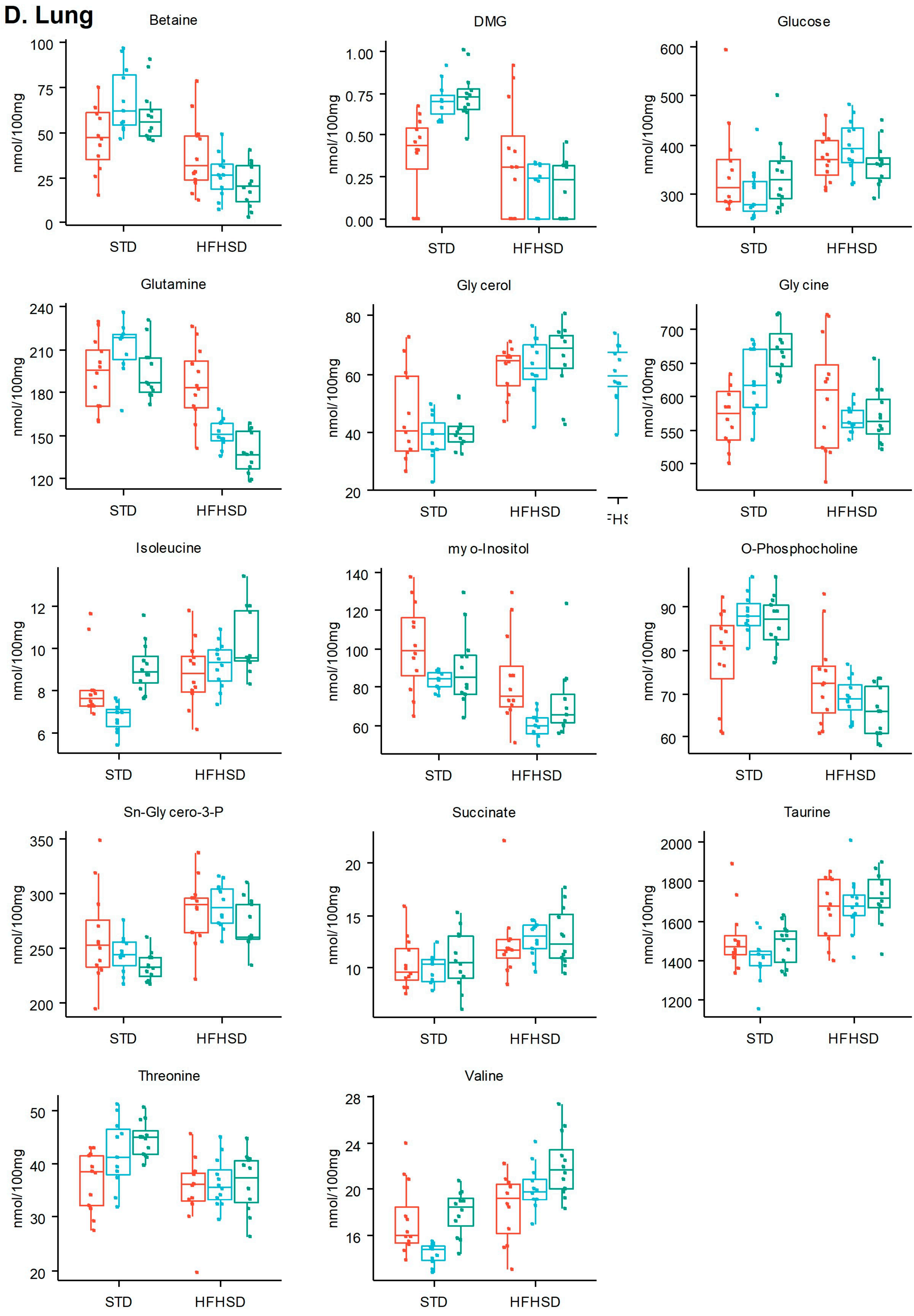

D. Lung
Betaine
DMG
Glucose
Glutamine
Gly cerol
Gly cine
Isoleucine
my o-Inositol
O-Phosphocholine
Sn-Gly cero-3-P
Succinate
Taurine
Threonine
Valine
nmol/100mg
STD
HFHSD

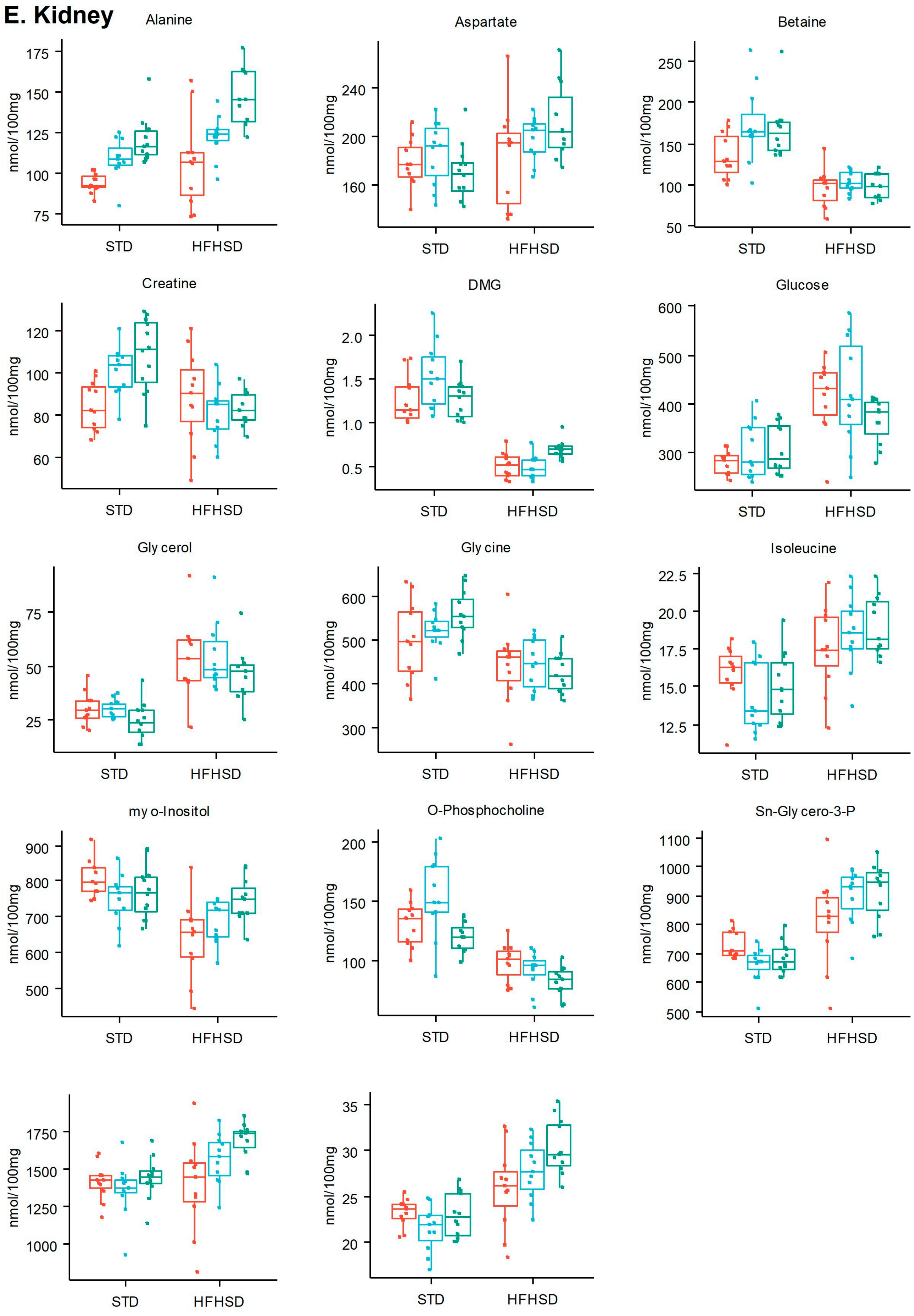
E. Kidney
Alanine
Aspartate
Betaine
Creatine
DMG
Glucose
Gly cerol
Gly cine
Isoleucine
my o-Inositol
O-Phosphocholine
Sn-Gly cero-3-P
nmol/100mg
STD
HFHSD

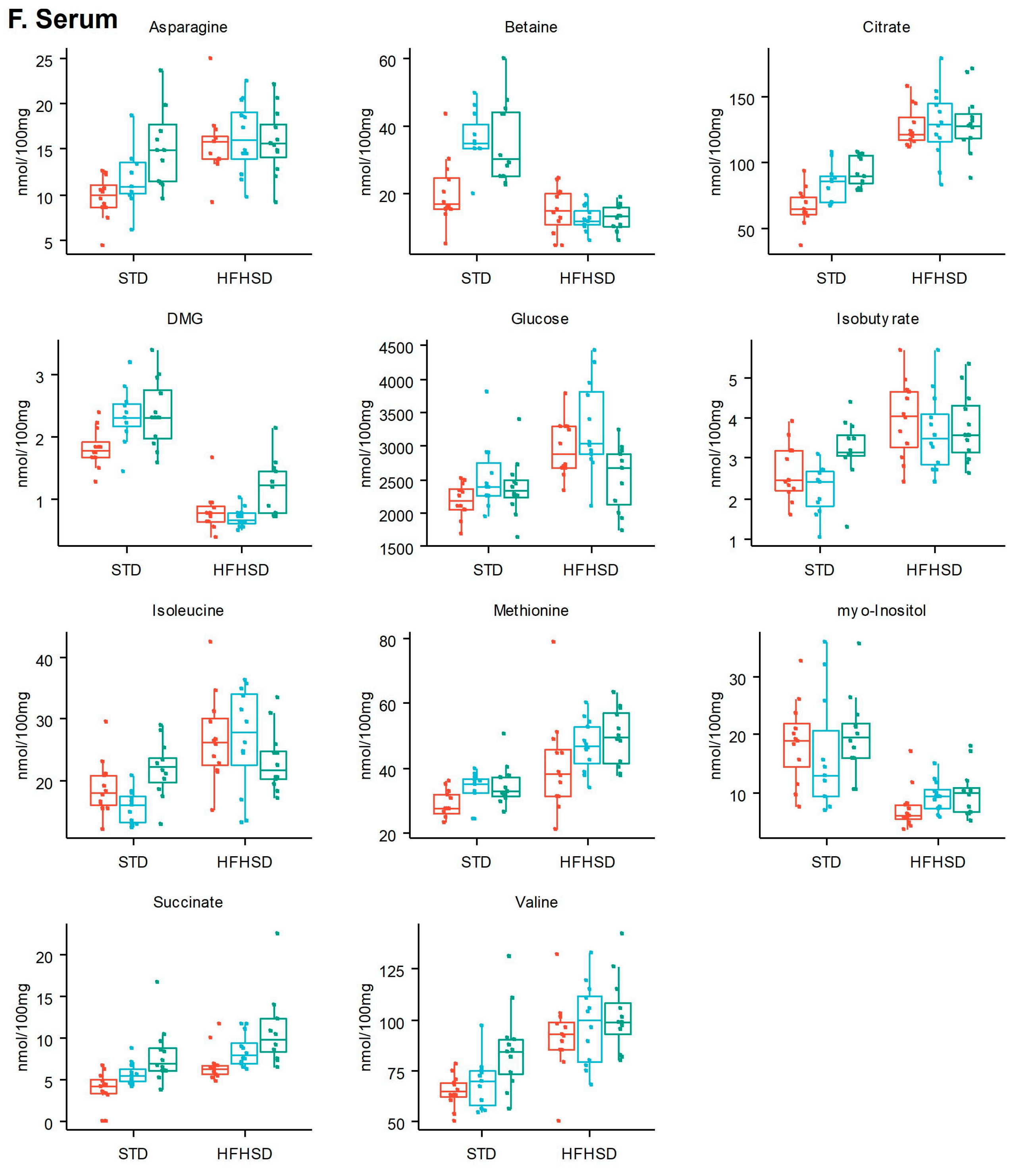


**Supplementary Figure 4.** Metabolite concentrations in nmol/100 mg of organ, depending on the diet (STD and HFHSD) and over time (6, 10 and 14 weeks respectively in red, blue and green). Only metabolites that present a significant impact of diet on ANOVA and t-tests are reported. All measurement points are represented on the box plots. The 3 horizontal bars represent the median, the first and the third quartile. The brain does not have any significant metabolites impacted by the diet. The metabolites impacted by the diet are reported in (A) for the heart, (B) kidney, (C) liver, (D) lung, (E) muscle, and (F) serum. The associated statistics are available in Table 1.
